# Does source credibility matter for point-of-decision prompts? A quasi-experimental field study to increase stair use

**DOI:** 10.1371/journal.pone.0225520

**Published:** 2019-11-21

**Authors:** Ivan P. Lee, Richard M. Walker

**Affiliations:** 1 Center for Experimental and Behavioral Public Administration, School of Public Affairs and Administration, Rutgers University–Newark, Newark, New Jersey, United States of America; 2 Chan Hon-pun Professor in Behavioural and Policy Sciences, Laboratory for Public Management and Policy, Department of Public Policy, City University of Hong Kong, Hong Kong, China; Universidad Autonoma de Madrid, SPAIN

## Abstract

A quasi-experimental field study was undertaken to examine whether the source credibility of point-of-decision (POD) prompts would affect their effectiveness in increasing stair use. POD prompts attributed either to a more credible source, a less credible source, or nothing were randomly installed in three student halls of residence at a public university in Hong Kong (plus a control). The stair and elevator use of residents were recorded by view-from-top surveillance cameras and counted using motion-detection software, resulting in 14,189 observations. The findings show that all the POD prompts can yield, as hypothesized, a significant positive effect on stair use. The relative increase in stair use was 2.49% on average. However, contrary to our second hypothesis, the POD prompt attributed to the more credible source was not the most effective intervention. The implications of these findings are discussed in conclusion.

## Introduction

Stairs are an environmental feature that can serve as an asset for health promotion. Taking the stairs is a practical form of moderate to vigorous physical activity that can be incorporated into everyday routines [1]. Stair use has a range of positive health effects, such as reduced low-density lipoprotein cholesterol, improved body composition, and muscle strengthening [2,3,4]. As such, an important public health initiative to date is to promote the accumulation of exercise in short bouts throughout the day by means of increasing stair use [5].

Point-of-decision (POD) prompts—messages positioned at the points-of-choice between methods of ascent or descent—are a widely adopted method to encourage individuals to use the stairs instead of the elevators or escalators [6,7,8]. Despite the converging evidence that POD prompts can encourage stair use in both public and workplace settings [9], there is still a persistent call for further exploration of designs that lend POD prompts to higher rates of effectiveness [1,10]. Yet, prior research has paid relatively little attention to the message characteristics and content of POD prompts, exploring how their changes may affect the interventions’ effectiveness. In the present study, we propose that the effectiveness of health-based POD prompts may be affected by a peripheral cue, namely, the source credibility of the messages. Specifically, we examine whether attributing POD prompts to a highly credible source could be a strategy for improving their effectiveness.

### Habitual behaviors, POD prompts, and stair use promotion

Habitual behaviors develop when people repeatedly perform a specific behavior (e.g., using the elevators or escalators) in a stable situation (e.g., where the elevators or escalators exist) to pursue their goals [6,11,12]. This co-occurrence between the situation and the behavior eventually creates a direct mental association between them, which will be strengthened each time the co-occurrence is repeated. Finally, this situation-behavior association is strengthened to the extent that when the situation is encountered, the behavior follows automatically and unintentionally [13,14,15].

Prior research has identified two major approaches for changing habitual behaviors. The first approach—*motivational interventions*—aims to induce people to change their behaviors motivationally and mindfully [6,16,17]. Grounded on the theory of planned behavior [18], such an approach includes interventions that make individuals contemplate attitudes, subjective norms, or perceived behavioral control, making them develop counter-habitual behavioral intentions and act accordingly [17,19].

The motivational approach alone is not always enough to break habits. It is because the activation of habitual behaviors on encountering situations occurs automatically without involving conscious deliberation. If the association between situations and behaviors remains unchanged, individuals may fail to activate prior counter-habitual intentions and hence keep their habits [20,21]. This is called *intention-behavior gap* [22]. In the case of stair use, individuals may walk straight into an elevator before their intention to be more physically active occurs to them. Hence, in addition to motivational interventions, it is suggested that a second approach, namely *volitional interventions*, is also important for changing habitual behaviors. Such an approach focuses on making individuals enact their prior counter-habitual intentions [6,16,17]. It includes interventions that remove critical stimuli from the environment that elicit habitual responses [11], and those adding new contextual cues to the situation that “remind” people of their counter-habitual intentions [6]. These interventions break the automatic link between situations and behaviors, narrowing down the intention-behavior gap.

In the context of stair use promotion, it has been argued that POD prompts are a combination of motivational *and* volitional approaches to induce behavioral change [6,23]. For the motivational aspect, POD prompts deliver health or other messages that are repeatedly read. Such a repeated exposure to the messages helps to improve attitudes, and hence intentions, towards stair use. For instance, prior qualitative research has indicated that some individuals do not consider stair use as a physical activity with health benefits [24]. Thus, a persuasive prompt that targets attitudes by specifying the benefits obtainable from stair use might increase individuals’ intentions to use the stairs [25]. For the volitional aspect, POD prompts change the contextual cues at the place where the habitual behavior occurs [12,25]. They provoke deliberation by people about using the stairs rather than choosing the escalators or elevators in a mindless manner. They also remind people of their prior counter-habitual intention and suggest an immediate opportunity for its fulfilment. Importantly, Lewis and Eves suggested that an effective POD prompt should contain both the motivational and volitional components [6]. Their study found no effect upon behavior when either intervention component was positioned alone. In contrast, the simultaneous positioning of both components increased stair use.

In the present study, we conceptualize POD prompts as motivational *and* volitional interventions that increase stair use through two mechanisms: 1) advising the benefits obtainable from stair use that help individuals to develop a counter-habitual intention, and; 2) providing contextual cues to narrow down the intention-behavior gap that helps individuals to enact their prior intention.

### The effectiveness of POD prompts and source credibility

Despite evidence that successful POD prompts could bring an increase in stair use ranged from 0.3 to 10.6% [9,26,27], there is still a persistent call for further exploration of designs that lend POD prompts to higher rates of effectiveness [1,10]. Jennings and colleagues suggested that the *type* of messages may matter: they find that time-based messages may be more effective than health-based messages in inducing behavior change in certain settings such as public transit [1]. However, there has been little systematic effort to examine whether the *peripheral cues* of POD prompts would affect their effectiveness. Theories of persuasion hold that the persuasiveness of a message could be significantly affected by its peripheral cues such as source and message characteristics [28,29]. Nevertheless, in the context of stair use interventions, only a handful of studies have examined whether the effectiveness of POD prompts will be affected by the variations in message characteristics. For example, only three relevant studies were listed in Bellicha and colleagues’ systematic review [9]. They include Cooley and colleagues’ and Russell and Hutchinson’s studies about positive versus negative-themes [30,31], and Lewis and Eves’s study about message complexity (i.e., simple versus complex messages) [32]. More research is therefore warranted to address this research gap, which could have important implications for revealing how messages should be tailored in order to maximize the positive effect of POD prompts.

Source credibility is one of the peripheral cues that could possibly matter for the effectiveness of POD prompts. Source credibility is concerned with the characteristics of the message sender that influence the receiver’s acceptance of the message communicated [33]. The dimensions of source credibility have been commonly identified to consist of expertise and trustworthiness [34,35,36,37]. *Expertise* refers to “the perceived ability of the source to make valid assertions” [37], that is, the extent to which the communicator is qualified to provide valid and accurate information or discuss a particular subject [35]. *Trustworthiness* is defined as “the perceived willingness of the source to make valid assertions” [37]. It refers to an audience’s belief that the communicator provides information in an honest, fair and sincere manner [33,38].

For decades, researchers in many fields have found that source credibility affects the persuasiveness of messages. A highly credible source commonly induces more persuasion towards the advocacy than a low-credibility one [34]. In the context of physical activity promotion, for example, research has reported that health promotion messages attributed to a highly credible source consistently produce more favorable attitudes, leading to stronger behavioral intentions and a greater participation in physical activity than messages attributed to low credible sources [39]. According to the Elaboration Likelihood Model [29], source credibility would have a more important influence under the condition of low involvement (or low elaboration). A low involvement condition could be caused by the low personal relevance between the message receivers and the issues, and by high environmental distractions (e.g., noise). Under the condition of low involvement, message receivers are less likely to scrutinize and think carefully about the messages. Instead, they tend to rely on peripheral cues such as the source credibility of messages to make their response.

### Hypotheses

In the present study, we hypothesize that the source credibility of POD prompts will affect their effectiveness in increasing stair use. It is because this factor may affect the motivational mechanism of POD prompts in bringing effects. As discussed above, an important component of POD prompts is to advise the benefits obtainable from stair use that helps individuals to develop a counter-habitual intention. Such a persuasive function of POD prompts is thus supposedly to be subject to the influence of source credibility. Besides, as POD prompts are typically positioned in open areas where individuals are less likely to engage in high elaboration, source credibility as a peripheral cue could be of particular relevance in such a setting.

In particular, we hypothesize that while POD prompts can increase stair use, the POD prompt attributed to a more credible source would be more effective than otherwise identical POD prompts that are attributed to a less credible source, or nothing. Two research hypotheses are formulated:

**Hypothesis 1 (H1)**: The presence of POD prompts will increase individuals’ likelihood to use the stairs instead of the elevators
**Hypothesis 2 (H2)**: A POD prompt attributed to a more credible source will be more effective in increasing stair use than prompts that are attributed to a less credible source, or nothing

## Materials and methods

To test these hypotheses, we conducted a quasi-experimental field study with a before and after control group design in four student halls of residence at a public university in Hong Kong. We manipulated the degree of source credibility of POD prompts across the three treatment locations and examined their differential effect, if any, on stair use as compared to the control location.

### Setting and participants

The experiment was conducted at the City University of Hong Kong (CityU). The climate of Hong Kong is subtropical with high temperatures and humidity levels. Prior research suggests that the rate of stair use in this city are lower than that in the U.S. and U.K., and the climate may be a barrier to lifestyle physical activity interventions [40].

The residence halls were built around the same time. They have a very similar infrastructure and foyer, and hence a similar staircase design and visibility. Each hall has 12 to 15 floors, and each floor contains 10 to 16 twin rooms. There are one staircase and two elevators (which are closely located) in each building. The front door of the halls is password and ID protected. Non-residents need to get permission before entering the buildings. This reduces the chance of treatment contamination between the experimental groups.

Each hall has around 350 residents, consisting of local (Hong Kong) and non-local, undergraduate and graduate, female and male students. Students are allocated to residences on a random basis if no specific preference is indicated during the application process. All residents in the treatment and control locations received a short email sent by CityU’s Student Residence Office two weeks before the commencement of the experiment. They were informed that additional surveillance cameras would be installed on the ground floor of their residence hall to record their stair and elevator use behaviors for academic purpose, but their facial details would not be identified. (The residence halls have already installed their own surveillance cameras on the ground floor for security purposes, but these original cameras could not record the stair/elevator use behaviors of the residents). They were not informed about the specific purpose of this study—that is, to study how POD prompts, and the differences in message source, may affect their stair use behavior. An ethics approval was obtained from CityU’s Human Subjects Ethics Sub-Committee before the commencement of the study (reference#: 2-4-201412). The requirement for informed consent was waived because researchers did not directly interact with individual subjects or collect personally identifiable information.

### Research design

**Fig 1:**
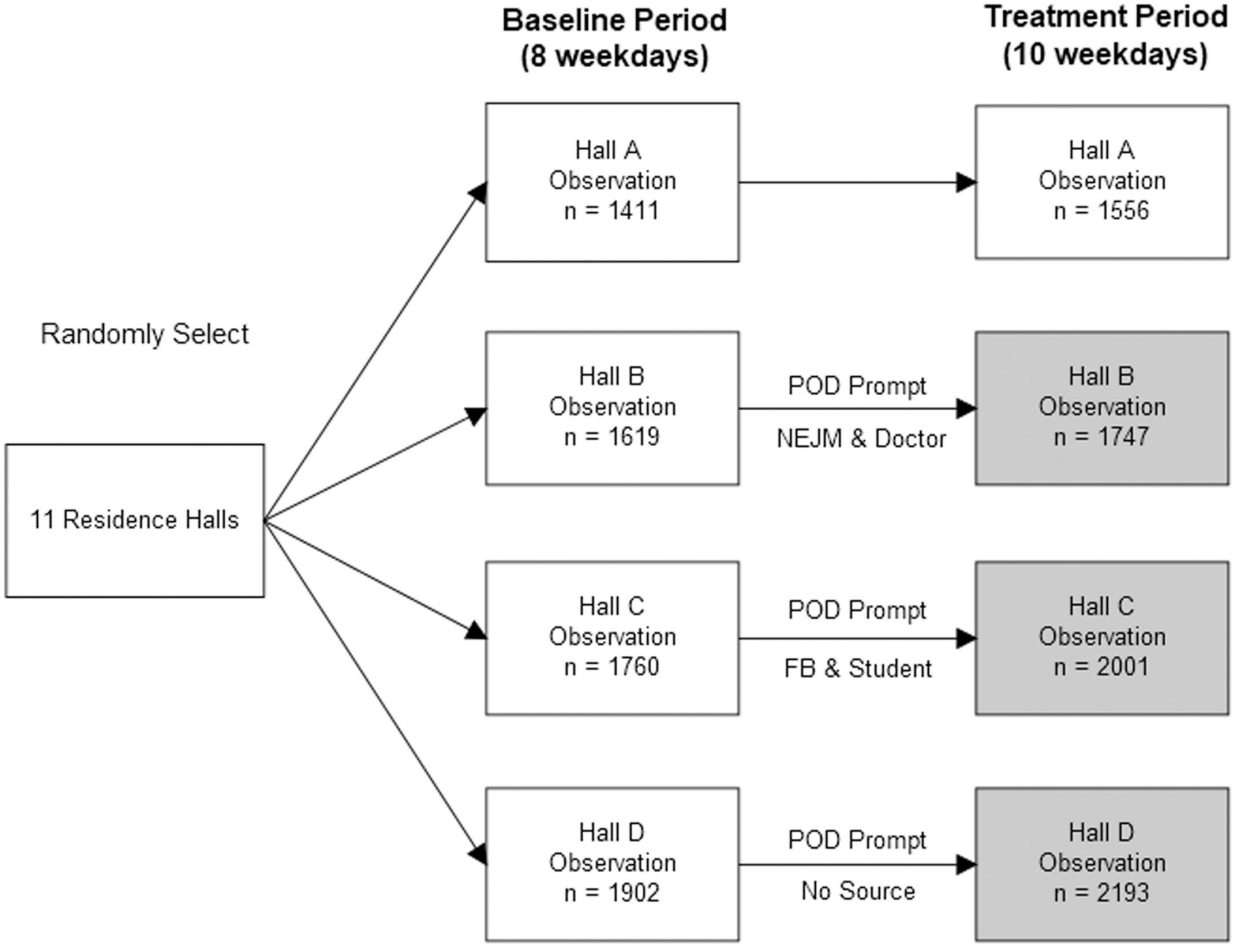
The research design. NEJM stands for New England Journal of Medicine. FB stands for Facebook.

Fig 1 illustrates the research design. Four out of eleven residence halls of the public university were randomly selected. Hall A was the control group which did not receive an intervention. Three halls formed the treatment groups: Hall B information attributed to a more credible source; Hall C received information attributed to a less credible source; and Hall D information without attribution to any source. The experiment was conducted from April 13 to May 6, 2016, in two time periods. The first time period was the baseline that lasted for 8 weekdays (the weekend was excluded from observation). The second was the treatment period where interventions were installed to the residence halls, which ran for 10 weekdays. The interventions were set up on the weekend after the baseline period (i.e., April 23).

### Intervention tools

A foamboard (60cm x 80cm) mounted with a 3-foot tripod was placed in each treatment hall (i.e., Hall B, C, D) between the staircase and the elevators at the foyer on the ground floor. On each upper floor, an A3 size poster (with an identical design to the foamboard, except an adjustment in the message; see below) was placed between the staircase and the elevators. (The setup of the interventions is shown in S1 Fig).

**Fig 2:**
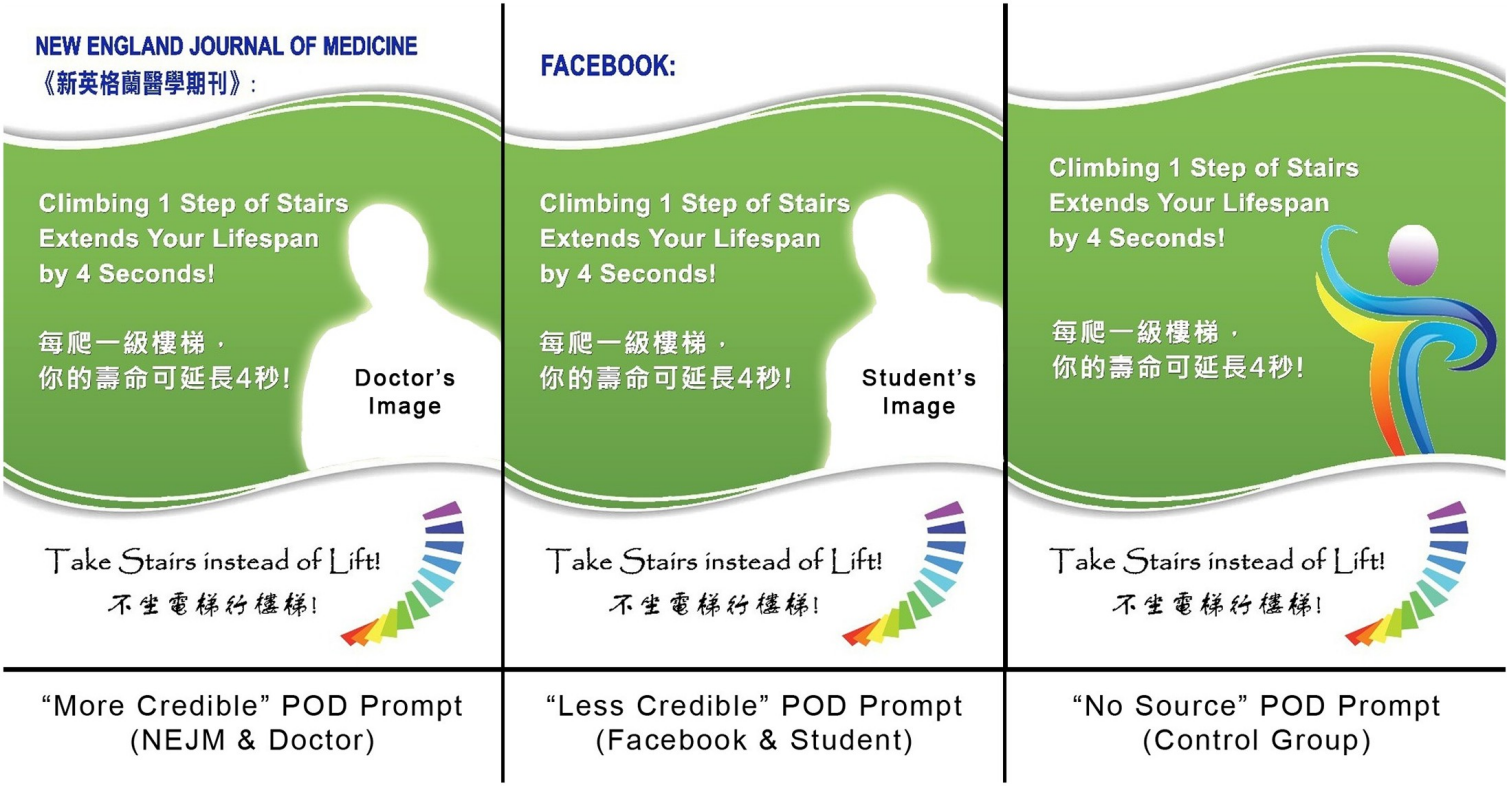
The design of intervention tools. This figure shows the design of the interventions placed on the ground floor. The message of the interventions on the upper floors is different: it becomes “Descending 1 Step of Stairs Extends Your Lifespan by 1.3 Seconds!”; The photos of the student and doctor are not shown in this article due to copyright restrictions. They were shown in the actual study.

Fig 2 shows the design of the POD prompts. It was a combined use of text and image, a design adopted by the majority of prior research on stair use interventions [1]. The interventions had 3 major components, namely, a standard message across all conditions, the source (varied by conditions), and an image (varied by conditions). First, the message was written as “Climbing 1 Step of Stairs Extends Your lifespan by 4 Seconds!” for the interventions on the ground floor (i.e., the foamboards). This message was the same for all conditions of source credibility. The source of this information was a letter published in the *New England Journal of Medicine (NEJM)* [41] and has appeared on *Facebook*. Based on the calculation suggested by the authors, and a theoretical calculation that the energy cost of stepping down is about 3 times lower than that of stepping up [42], we converted the message to “Descending 1 Step of Stairs Extends Your Lifespan by 1.3 Seconds!” for the interventions on the upper floors (i.e., the A3 size posters). This health message was selected by the participants of two focus groups (n = 12 and n = 8) conducted in 2015. The first group consisted of students from the same public university who did not live in the residence halls. The second groups consisted of local citizens from a public housing estate near the university. The participants suggested that this health message seems to be more effective than other alternatives in encouraging stair use. However, they also suggested that the persuasiveness of such a message is sensitive to the credibility of the source.

Second, the source of the message was the *NEJM* in the “more credible” treatment group. It was changed to *Facebook* in the “less credible” treatment group and was omitted in the “no source” treatment group. The *NEJM* and *Facebook* were significantly different in terms of their level of credibility. This difference was verified in an online pretest. Specifically, in a within-subject repeated measures experiment, a separate group of students (n = 94) from the same university who did not live in the residence halls were recruited. Each of them was randomly presented with two out of six health messages attributed either to *NEJM*, to *Facebook*, or to other media *sources*. They were asked to rate the credibility level of the sources using an 8-item perceived credibility scale. (The question and the scale are shown in the S1 Appendix). Most of the scale’s items were adopted from the study of Hellmueller and Trilling [43], which provides a systematic review of source credibility measures in communication journals from 1951 to 2011. The Cronbach’s alpha of this scale was 0.94. A total of 188 observations were yielded, containing 36 observations for *NEJM* and 32 observations for *Facebook*. The results show that the credibility score of *NEJM* was significantly higher than that of *Facebook* (M_NEJM_ = 4.4, SD = 1.38 vs. M_FB_ = 3.35, SD = 1.18; t(66) = 3.37, *p* = 0.001, *d* = 0.82).

Third, a white male doctor’s image was attached to the POD prompt in the “more credible” treatment group. A white male student’s image was attached to the “less credible” treatment, and an abstract figure was attached to the “no source” treatment. The doctor and the student were perceived to be different in terms of their level of credibility. Such a difference was also verified in the online pretest illustrated above. In particular, after evaluating the credibility of the media sources, each subject was randomly presented with three out of nine messages attached either to the doctor’s image, to the student’s image, or to the images of other persons. They were then asked to rate the credibility level of the persons using a credibility scale with another 8 items (see S1 Appendix). These items were adopted from the study of Ohanian [44], which are applicable for assessing individual-level credibility. The Cronbach’s alpha of this scale was 0.95. A total of 282 observations were yielded, containing 35 observations for the doctor and 28 observations for the student. The results show that the credibility score of the doctor was significantly higher than that of the student (M_Doctor_ = 5.19, SD = 1.18 vs. M_Student_ = 3.82, SD = 0.75; t(61) = 5.35, *p* < 0.001, *d* = 1.36).

### Measures

The dependent variable of this research is stair use, which combines ascent and descent. It is a dichotomous variable where 0 represents the observation when a person used the elevators, and 1 represents the observation when a person used the stairs. This measurement considered only the residents’ movement from the ground floor to the upper floors, or from the upper floors to the ground floor. It did not include the behaviors of people who travelled between the upper floors (e.g., traveling from 10/F to 6/F). However, the amount of this kind of travelling behaviors should be trivial in the resident halls, since there is no special function room in the floors above the ground floor.

The stair and elevator use of the residents were recorded by view-from-top surveillance cameras mounted vertically on the foyer’s ceiling on the ground floor in the experimental halls. There were 3 cameras installed in each hall, recording the usage of one staircase and two elevators respectively. For reasons of privacy, the facial details of the residents, as well as their gender, were not identified. (The setup of the view-from-top surveillance cameras is shown in S2 Fig).

All video records made between 10.00–11.00, 14.00–15.00, and 17.00–18.00 on the weekdays were watched. The frequencies of people using the stairs and the elevators were counted by a team of student research assistants with the support of a motion detection software called SmartMotion [45]. The software captured events from video footage, filtering a long video into multiple short clips containing human movements. The research assistants then looked at these short clips and counted the total frequencies of stair use and elevator use. A part of the video clips was watched and counted by all research assistants. The error variance of their counting results is below 5% of the total counts. A total of 14,189 observations were coded.

Notably, like some prior research conducted in field settings [46, 47], the present study did not recorded the total number of people actually involved in the experiment, the demographic information of the subjects, and their attention and attitudes towards the treatments. The focus of the research is to examine the casual relationship between the interventions and the observed behavioral outcomes.

### Statistical analysis

Like the prior studies on stair use [7,8], the unit of analysis of the present study is the observation of human behaviors (i.e., stair use and elevator use). To test hypothesis 1, we used logistic regression [7] and a difference-in-differences (DID) approach to estimate how the odds of stair use changed from the baseline to the treatment period in the treatment groups (i.e., Hall B, C, D) relative to that in the control group (i.e., Hall A). The DID approach estimated the relative change in stair use over time associated with the POD prompts as the difference of 2 differences: the difference in stair use between the treatment and control groups, and the difference in stair use before and after the POD prompts were installed. This approach was adopted because the experiment was a quasi-experiment with a controlled before and after design. The regressor of interest varied only at an aggregate level (i.e., the residence halls), and we assumed that there is a parallel trend between the halls in the treatment and control groups over time. We made this parallel trend assumption because the residence halls are closely located at the same university. If there is any potential factor (other than the treatments) that may influence stair use, all the residence halls should be jointly affected. In this sense, there should be a parallel trend in stair use between the residence halls involved in this study. All these prerequisites make the DID approach applicable in this research [48].

To test hypothesis 2, we compared the DID estimator of the effect of different treatments in the model illustrated above—that is, we compared the magnitude of the relative change in stair use in different treatment groups. In addition, we also made a direct comparison between the treatments, using the DID approach to estimate how the odds of stair use changed from the baseline to the treatment period in the “more credible” treatment group (i.e., Hall B) relative to that in the other treatment groups (Hall C and Hall D). However, the results of this direct comparison need to be interpreted with caution, since there is a debate on whether the DID estimation can be applied to the comparison between multiple treatments [49,50].

## Results

The descriptive statistics and the difference-in-difference estimates are presented in Table 1, and the logistic regression results are presented in Table 2. We regressed the dependent variable (i.e., stair use) on a dichotomous variable indicating the experimental period (i.e., treatment vs. baseline period), a nominal variable indicating the experimental locations (i.e., treatment vs. control locations or Hall B, C, D vs. Hall A), and the interaction between these variables (i.e., the DID estimator). Model 1 of Table 2 presents the combined results of all treatment groups, while Model 2 looks at each treatment group separately.

**Table 1:** Stair use in the treatment and control groups: Descriptive statistics and difference-in-differences estimates.

	Baseline Period	Treatment Period	Difference (treatment vs. baseline period)
Control Group (Hall A)	4.04	2.19	-1.85**
Treatment Groups (Hall B, C, D)	3.96	4.6	0.64
Difference (treatment vs. control groups)	-0.08	2.41**	2.49**
Individual Treatment Group			
Hall B (NEJM & Doctor)	4.26	4.01	-0.25
Difference (Hall B vs. control group)	0.22	1.82**	1.6*
Hall C (FB & Student)	4.6	6.15	1.55*
Difference (Hall C vs. control group)	0.56	3.96***	3.4***
Hall D (No Source)	3.1	3.65	0.55
Difference (Hall D vs. control group)	-0.94	1.46*	2.4**

*Note*: The regular numbers are the percentage (and the difference in percentage) of stair use, where the percentage was calculated by [stair use / (stair use + elevator use)*100%]; the underlined and bolded numbers are the difference-in-differences estimation.

*p < .05,

**p < .01,

***p < .001

**Table 2:** Stair use in the treatment and control groups: Logistic regression results.

Variable	Model 1		Model 2	
	OR	95% CIs	OR	95% CIs
Treatment Period (Ref: Baseline)	0.53**	0.34–0.82	0.53**	0.34–0.82
Treatment Groups (Ref: Control Group)	0.98	0.73–1.32		
Hall B—NEJM & Doctor			1.06	0.74–1.51
Hall C—FB & Student			1.15	0.81–1.62
Hall D—No Source			0.76	0.52–1.10
Treatment Period*Treatment Groups	2.20***	1.38–3.52		
Treatment Period*Hall B			1.77*	1.02–3.06
Treatment Period*Hall C			2.56***	1.52–4.30
Treatment Period*Hall D			2.23**	1.29–3.86
Constant	0.04***	0.03–0.05	0.04***	0.03–0.05
Observations	14,189		14,189	

*Note*: Odds ratios (OR) and 95% confidence intervals (CIs) of treatment period, treatment groups, and their interactions for stair use.

*p< .05,

**p < .01,

***p < .001

As shown in Table 1, in the baseline period, the percentage of stair use in Hall A (the control group) was 4.04%, compared to 4.26% in Hall B (the “more credible” treatment group), 4.6% in Hall C (the “less credible” treatment group), and 3.1% in Hall D (the “no source” treatment group). The results of logistic regression (not shown in the table) indicate that there is no statistically significant difference in stair use between the control and treatment groups in the baseline period (Hall A vs. Hall B: *p* = 0.760; Hall A vs. Hall C: *p* = 0.441; Hall A vs. Hall D: *p* = 0.148). This means that the 4 residence halls were arguably comparable in a quasi-experimental design despite the absence of a randomization process at the individual level.

**Fig 3:**
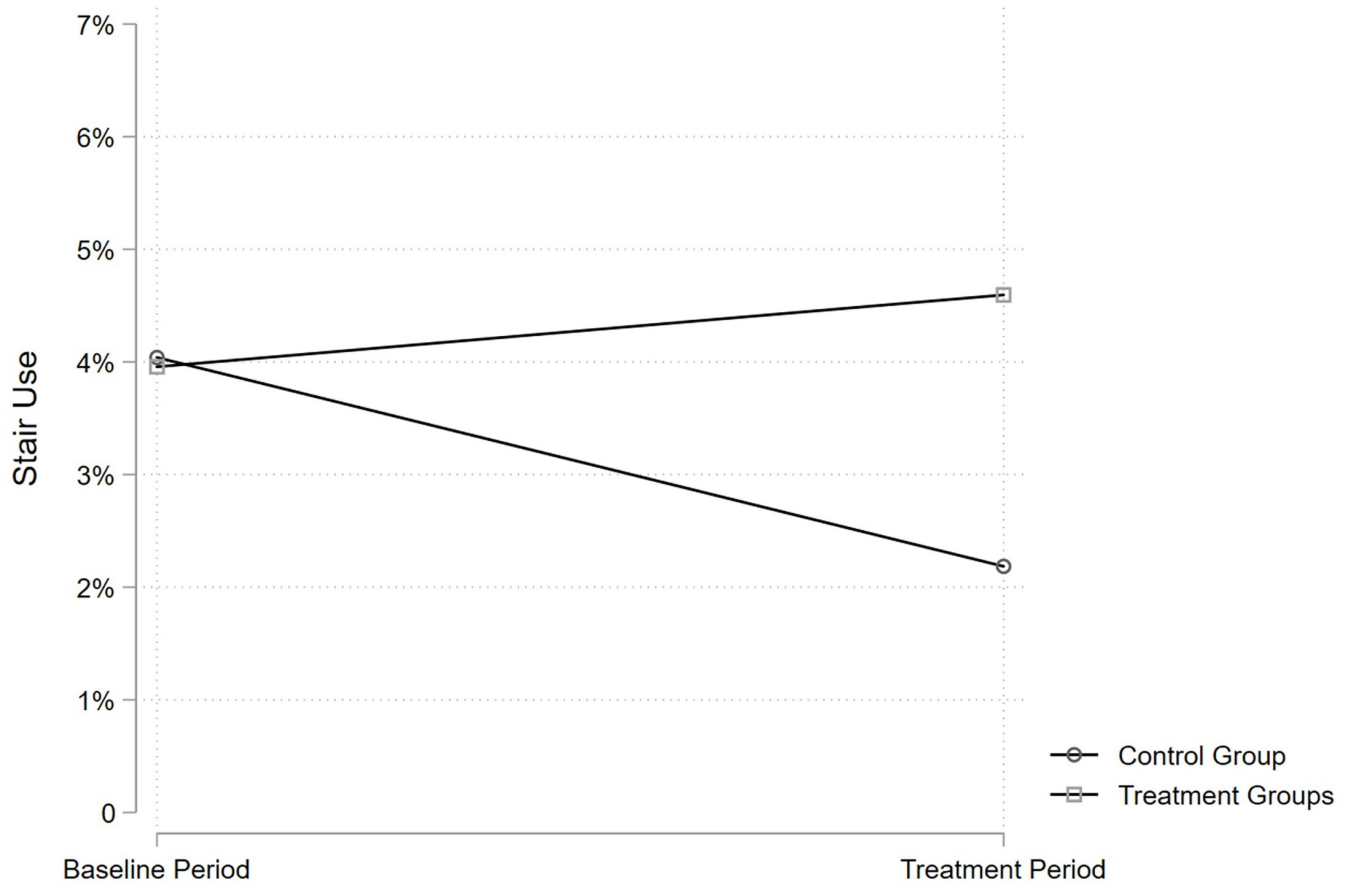
The percentage of stair use in the treatment and control groups in the baseline and treatment period. The percentage of stair use was calculated by [stair use / (stair use + elevator use) *100%].

In the treatment period, the percentage of stair use in the control group dropped from 4.04% to 2.19%, and this decline is statistically significant (OR = 0.53, 95% CI = 0.34–0.82, *p* = 0.004), as seen in Table 2. This means that there was a trend of decreasing stair use during the experimental periods (see below for a discussion of the potential reasons), and we assume that this trend could be observed in all the residence halls if the treatments were absent (i.e., the parallel trend assumption). However, as shown in Tables 1 and 2, the percentage of stair use in the treatment groups increased over time. The relative increase in stair use (i.e., the DID estimator) was 2.49%, which is statistically significant (OR = 2.20, 95% CI = 1.38–3.52, *p* = 0.001). A counter-trend treatment effect was observed as stair use increased. Fig 3 visually displays these results.

Concerning each individual treatment group, the results show that all the interventions could yield a significant counter-trend effect in affecting stair use. In particular, as seen in Tables 1 and 2, the relative increase in stair use was 1.6% in Hall B with the “more credible” treatment (OR = 1.77, 95% CI = 1.02–3.06, *p* = 0.042), 3.4% in Hall C with the “less credible” treatment (OR = 2.56, 95% CI = 1.52–4.30, *p* < 0.001), and 2.4% in Hall D with the “no source” treatment (OR = 2.23, 95% CI = 1.29–3.86, *p* = 0.004). Surprisingly, the “more credible” treatment seemed to be the least effective one, while the “less credible” treatment seemed to be more effective, as the latter brought a larger increase in stair use over time relative to that in the control group.

**Fig 4:**
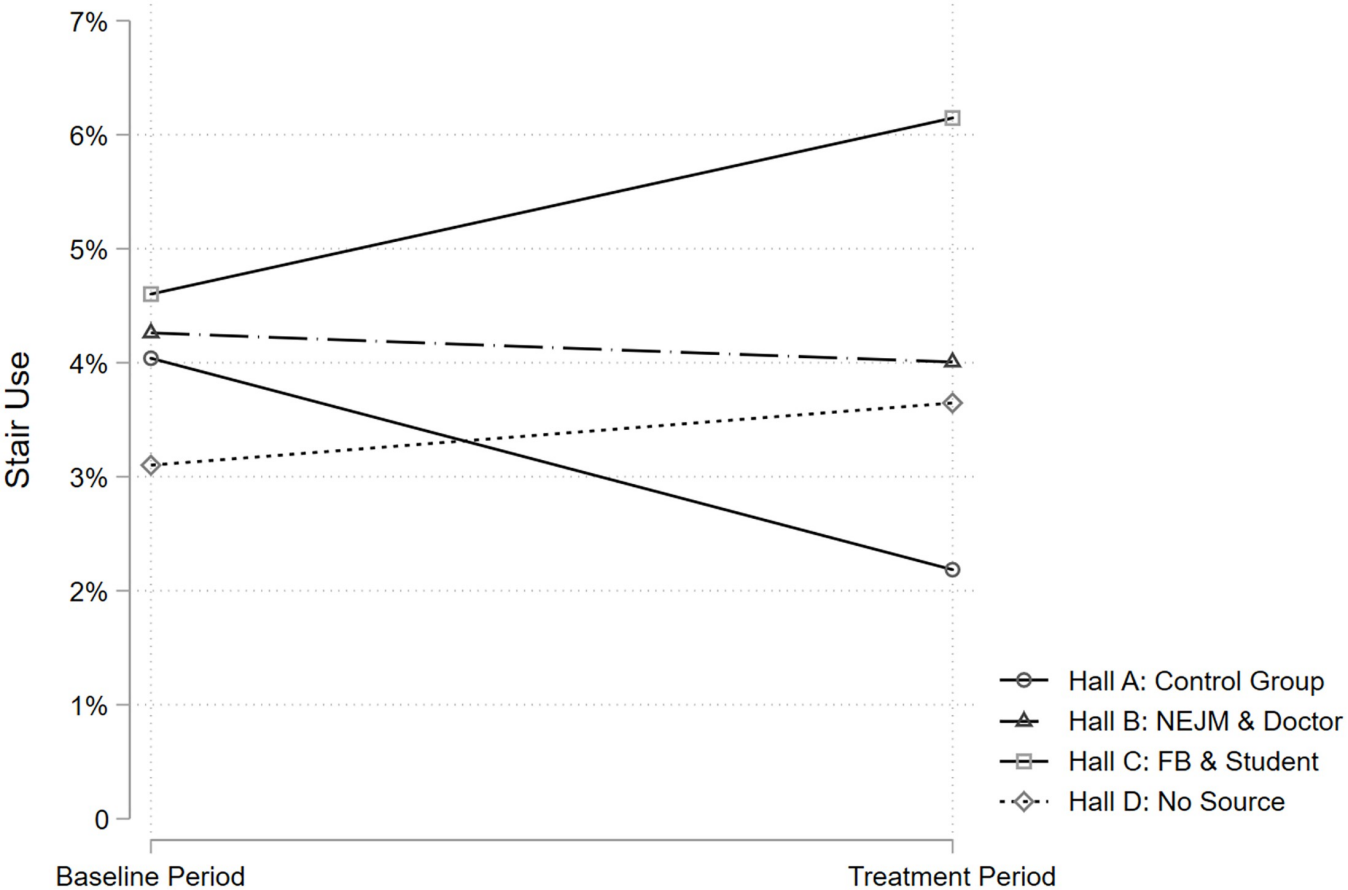
The Percentage of Stair Use in Each Residence Hall in the Baseline and Treatment Period. The percentage of stair use was calculated by [stair use / (stair use + elevator use) *100%]; NEJM stands for New England Journal of Medicine, and FB stands for Facebook.

Table 3 show the results of the direct comparison between treatments. The DID estimators are statistically insignificant (Treatment Period*Hall C: *p* = 0103; Treatment Period*Hall D: *p* = 0.345). This means that the change in stair use over time in Hall B associated with the “more credible” treatment did not significantly differ to that in the Hall C and D associated with other treatments. Fig 4 graphs these findings.

**Table 3:** Stair use in the treatment groups: Logistic regression results.

Variable	OR	95% CIs
Treatment Period (Ref: Baseline)	0.94	0.67–1.32
Treatment Groups (Ref: Hall B)		
Hall C—FB & Student	1.08	0.78–1.51
Hall D—No Source	0.72	0.50–1.02
Treatment Period*Treatment Groups		
Treatment Period*Hall C	1.45	0.93–2.26
Treatment Period*Hall D	1.26	0.78–2.04
Constant	0.04***	0.03–0.05
Observations	11,222	

*Note*: Odds ratios (OR) and 95% confidence intervals (CIs) of Treatment period, Treatment groups, and their interactions for stair use.

*p< .05,

**p < .01,

***p < .001

## Discussion

The percentage of stair use in the control group (Hall A) dropped statistically significantly from 4.04% in the baseline period to 2.19% in the treatment period, suggesting that there was a trend of decline in stair use. One of the potential reasons for this trend is that the weather in Hong Kong gets warmer during April and May. According to the Hong Kong Observatory, the average of the daily mean temperatures was 22.4 degree Celsius in the baseline period and was 26.5 degree Celsius in the treatment period [51]. The increasing temperatures might undermine the people’s willingness to use the stairs.

Examining how weather conditions may affect stair use is beyond the scope of this research. In the present study, we used a before and after control group design and a DID approach to control the influence of time varying factors (e.g., the weather conditions) and examine how the POD prompts may increase stair use. The results show that all the POD prompts—either attributed to a more credible source, a less credible source, or nothing—can yield a significant counter-trend effect in affecting stair use. The relative change in stair use over time associated with the treatments is 2.49%. This amount is within the range of effectiveness (i.e., 0.3 to 10.6%) reported in previous studies [26,27]. These results support the first hypothesis of the present study: the presence of POD prompts will increase individuals’ likelihood to use the stairs instead of the elevators.

The second hypothesis concerning the influence of source credibility is not supported. Although the POD prompt attributed to the more credible source had a counter-trend effect in affecting stair use (1.6% relative change), the positive effect associated with this treatment was smaller than that associated with the “no source” treatment (2.4% relative change) and those attributed to the “less credible” source (3.4% relative change), but the differences between these effects are not statistically significant. A POD prompt attributed to a more credible source is not more effective in increasing stair use than prompts that are attributed to a less credible source, or nothing.

### Why might source credibility not matter for POD prompts?

The second part of the findings illustrated above is inconsistent with the traditional understandings that messages attributed to a more credible source are more effective in producing attitudinal and behavioral changes than those attributed to a less credible one [34,39]. Why might source credibility, at least in our empirical case, not matter for the effectiveness of POD prompts? Three possible explanations are suggested.

First, since the installation of interventions were authorized by the Student Residence Office of the university, the residents might have assumed that the approved messages were credible at the outset. In this circumstance, attributing the POD prompt to a highly credible source (NEJM & Doctor) might contribute little to further increase its level of credibility, while attributing it to a less credible source (Facebook & Student) might not significantly undermine its credibility level. As a result, the expected impacts of source credibility cannot be observed, since the treatments might indeed be perceived as similarly credible.

Second, it is possible that the residents might not pay attention to the nuances of the POD prompts—that is, they might not actually recognize the sources attributed to the POD prompts. In other words, there might exist an issue of treatment noncompliance. In this circumstance, attributing POD prompts to different sources *per se* might have little impact to their effectiveness.

Third, it is also possible that the residents might have prior attitudes towards stair use before receiving the treatments, and the presence of prior attitudes might have undermined the effect of source credibility. Over the past two decades, government agencies and public health professionals around the globe have made enormous efforts to promote the health benefits of using the stairs. In Hong Kong, for example, the Department of Health (DoH) have disseminated health guidelines and periodically launched campaigns to promote stair use in workplace and housing blocks [52]. Thanks to these efforts, the residents might have knowledge about the health benefits and established positive attitudes towards stair use prior to the interventions. Research suggests that the credibility of external sources is particularly influential when one is unable to access a prior attitude about an object and does not construct a personal attitude towards it based on, for example, the message content [53,54]. However, if people have established attitudes about the object, those attitudes are likely to provide readily available, subjectively valid bases for a current evaluation of the object [55,56,57,58,59,60]. In this circumstance, the effects of source credibility will decay rapidly [61].

### Alternative factors

If the second reason presented above is true, it may imply that attitudinal strategies that seek to better advise the health benefits of stair use are not effective stair use interventions. This is because people in certain settings, such as university areas, may already have sufficient knowledge about the benefits of using the stairs. Strategies to improve the effectiveness of stair use interventions in these settings may have to consider using an alternative motivational approach. For example, prior research finds that time-based POD prompts are more effective than health-based messages in inducing behavior change in certain places [1]. This may imply that the effect of the motivational component of POD prompts can be improved through an *issue framing* strategy [62], which emphasizes other positive attributes of stair use (e.g., time-efficiency) rather than its health benefits.

In our study, the relative change in the percentage of stair use in the residence hall receiving the “Facebook & Student” treatment (3.4%) was larger than that in the hall receiving the “NEJM & Doctor” treatment (1.6%). Although the difference between the effectiveness of these treatments is statistically insignificant (OR = 1.45, 95% CI = 093–2.26, *p* = 0.1003), it still reveals that there could be potential alternative factors that may moderate the effectiveness of POD prompts. Our experiment did not intentionally manipulate these factors. But if they are targeted and used for tailoring the peripheral cues or the main content of POD prompts, the interventions’ effectiveness may be significantly improved.

One of these potential factors is social norms. *Social norms* refer to the beliefs that individuals hold about what the majority of other people do or approve of doing [63]. There are two major types of social norms, namely *descriptive norms* and *injunctive norms*. The former refers to individuals’ beliefs about the prevalence of a behavior within a group, while the latter refers to individuals’ beliefs about the extent to which others within the group would socially approve of us if we engaged in a particular behavior [64,65]. Research has shown that individuals are susceptible to social normative information in situations such as their home or campus residence hall [64,66,67], and the social norms approaches are effective in inducing behavior changes [67,68,69].

Although the “Facebook & Student” treatment in our experiment did not contain a social normative appeal, it might implicitly signal that the behavior of stair use, or more broadly speaking becoming physically active, are prevalent or socially approved among the peer group of students. As a result, the effectiveness of the health message in this treatment group might have been slightly boosted by this implicit factor, leading to the observed counterintuitive pattern: the “less credible” treatment seem to be more effective than the “more credible” one.

### Limitations

The first limitation of the present study is that the pretest did not evaluate and compare other characteristics of the sources and images. It is therefore unclear whether the two sources (NEJM vs. Facebook) and two images (doctor vs. student) are different in terms of their social normative influence and other alternative factors. In this sense, the present study can only demonstrate that a higher level of source credibility did not lead to a more effective POD prompt in a university setting. It cannot provide empirical evidence to show what exactly the “Facebook & Student” treatment contained that might potentially lend POD prompts to a higher rate of effectiveness. Future research is therefore warranted to address this question.

Second, like some prior research conducted in field settings [46, 47], the present study did not record the total number of people actually involved in the experiment, the demographic information of the subjects, and their attention and attitudes towards the treatments. Since there is no psychological measurement in the individual level, it is unclear whether and how the individual variables such as attitudes were affected by the POD prompts, and whether the increase in stair use was caused by the variations in these variables. It is also unclear whether the effectiveness of the POD prompts was reduced with repetitive interactions with them (e.g., after seeing it 1–2 times people might ignore the prompts). Besides, since the demographic information of the residents was not collected, it is unclear whether the treatment effect was heterogeneous across subjects with different socio-demographic status such as gender, age, exercise times per week, as well as the floor they were living on. That being said, our study provides important causal evidence showing how the placement of the POD prompts in a naturalistic setting affected individuals’ behaviors. It can shed lights on future research which may include measurements to trace individual level dynamics.

Third, the quasi-experimental design of this study did not randomly assign subjects to the experimental groups. Although the process of allocating students to residences and the similar infrastructure of the buildings make the groups largely comparable, there may be still some unobserved confounding factors that may threaten the internal validity of the study. A randomized controlled trial is therefore recommended in future research.

Fourth, we suggest that there was a trend of decline in stair use during the experimental period, and this trend was evident by the fact that stair use declined in the control condition (Hall A). We suggest that this trend was parallel across different experimental locations, since it may be caused by potential factors—such as warmer weather—affecting all the locations. However, it is possible that this decline was indeed caused by some unknown factors specific of Hall A, and the trend of change in stair use was not parallel across the experimental locations. In this case, the conclusions of this study might not reflect a true effect of POD prompts. We again recommend a randomized controlled trial in future research to rule out potential confounds.

Last, the post-treatment observation period of this study only lasted for 10 days. It is thus unclear whether and how long the short-term effect of POD prompts could sustain. Future research may extend the duration of the observation period to examine whether and how POD prompts work in the long-term.

### Implications

Keeping these limitations in mind, the present study has important implications for research and practice. For theory, our research corroborates the call for further exploration of how POD prompts should be tailored to specifically match the desired settings [1]. Conventional POD prompts are typically seen as volitional interventions for action initiation in that their function is to convert prior intentions into behavior [6]. Relatively less attention has been paid to the motivational component of POD prompts, exploring how the changes in message characteristics and content may affect the interventions’ effectiveness. Against this backdrop, our study suggests that in certain settings, like the university areas, the effect of health-based POD prompts may not be improved by better advising the health benefits of stair use. Rather, using the social normative approaches may be a better option. These preliminary results provide a foundation for further studying the motivational mechanism of POD prompts.

Practically, our study suggests that practitioners need to think deliberately and experiment with different ways to find out which solution works best. There is no “silver bullet” that can resolve all problems in all contexts. Our results show that even a well adopted classic communication strategy (i.e., attributing the message to a highly credible source) may lose its effectiveness under certain situations. It is therefore important to tailor the message content for different communities so that they can better suit the targeted groups’ motivations and experiences. For example, in the university areas, the messages from student representatives may work better than those from professionals, while in the other settings it may work the other way around.

Indeed, point-of-decision (sometimes called as point-of-action, point-of-choice, or point-of-purchase) interventions have been widely applied to a range of applied topics, including obesity prevention [70,46] and recycling [71]. Each of these behaviors is affected by a different set of motivations. In order to maximize the effectiveness of these interventions to improve people’s well-being, more efforts in information tailoring are required.

## Conclusion

People are regularly faced with choices about using the stairs or an elevator to move around buildings. The use of the stairs has been shown to have a range of health benefits. This study highlights how POD prompts can influence the choices people make and increase the use of stairs over the elevators. Our study also suggests that the impact of source credibility of the information provided in a POD prompt is contingent on the environment in which it is placed. Practically we conclude that POD prompts should be used to boost stair use. However, future research is required to ascertain the effectiveness of different prompts in different locations.

## Supporting information

S1 Appendix The questions in the pretest. (PDF)

S1 Fig The setup of the POD prompts. (TIF)

S2 Fig The setup of the view-from-top cameras. (TIF)
