# Supplementary material for: Does source credibility matter for point-of-decision prompts? A quasi-experimental field study to increase stair use
Source: PLoS One. 2019 Nov 21;14(11):e0225520. doi: 10.1371/journal.pone.0225520 (PMC6872137; doi:10.1371/journal.pone.0225520)
Supplement: S1 Appendix — (PDF) [file pone.0225520.s001.pdf]

S1 Appendix. The questions in the pretest

【Climbing one step of stairs extends your lifespan by 4 seconds】
【每爬樓梯一級，你的壽命可延長 4 秒】

This message is reported by:

New English Journal of Medicine
(新英格蘭醫學期刊)

I think this source is / I think:

	1	2	3	4	5	6	7	
Little Known	❍	❍	❍	❍	❍	❍	❍	Well known
Untrustworthy	❍	❍	❍	❍	❍	❍	❍	Trustworthy
Unauthoritative	❍	❍	❍	❍	❍	❍	❍	Authoritative
Unqualified	❍	❍	❍	❍	❍	❍	❍	Qualified
Biased	❍	❍	❍	❍	❍	❍	❍	Unbiased
Inaccurate	❍	❍	❍	❍	❍	❍	❍	Accurate
I would not accept	❍	❍	❍	❍	❍	❍	❍	I would accept what it says
I would not recommend	❍	❍	❍	❍	❍	❍	❍	I would recommend what it says to others

【Climbing one step of stairs extends your lifespan by 4 seconds】
【每爬樓梯一級，你的壽命可延長 4 秒】

This message is presented by:

Person's Image
(Doctor/Student)

I think this person is:

	1	2	3	4	5	6	7	
Unqualified	❍	❍	❍	❍	❍	❍	❍	Qualified
Unreliable	❍	❍	❍	❍	❍	❍	❍	Reliable
Unsympathetic	❍	❍	❍	❍	❍	❍	❍	Caring
Inexperienced	❍	❍	❍	❍	❍	❍	❍	Experienced
Insincere	❍	❍	❍	❍	❍	❍	❍	Sincere
Unattractive	❍	❍	❍	❍	❍	❍	❍	Attractive
Not an Expert	❍	❍	❍	❍	❍	❍	❍	An Expert
Untrustworthy	❍	❍	❍	❍	❍	❍	❍	Trustworthy

Note: Some subjects were randomly assigned to read the message from *Facebook* instead of *NEJM*. Likewise, some subjects were randomly assigned to evaluate either the image of the doctor or the student.
