# Supplementary figures and images for: Does source credibility matter for point-of-decision prompts? A quasi-experimental field study to increase stair use
Source: PLoS One. 2019 Nov 21;14(11):e0225520. doi: 10.1371/journal.pone.0225520 (PMC6872137; doi:10.1371/journal.pone.0225520)

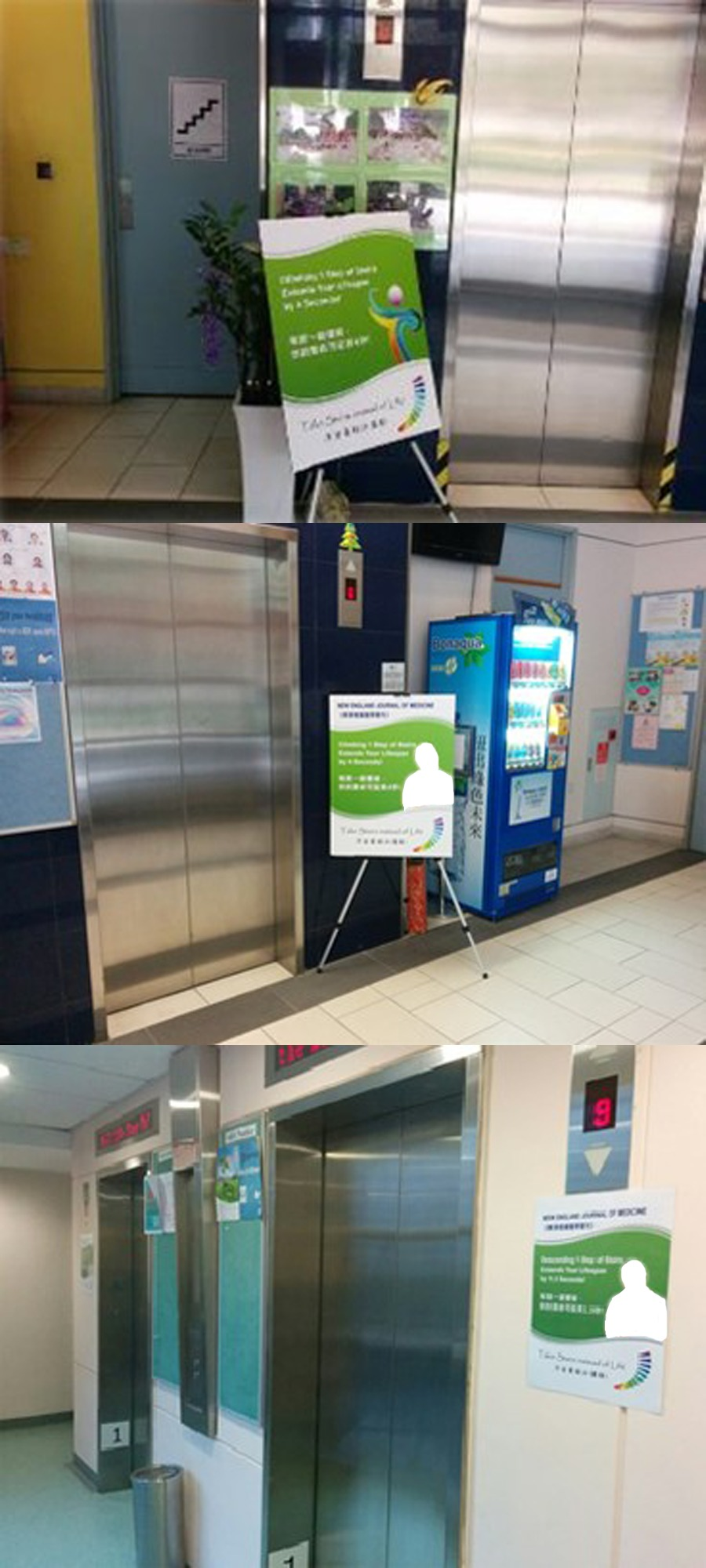

Supplement: S1 Fig — (TIF) [file pone.0225520.s002.tif]

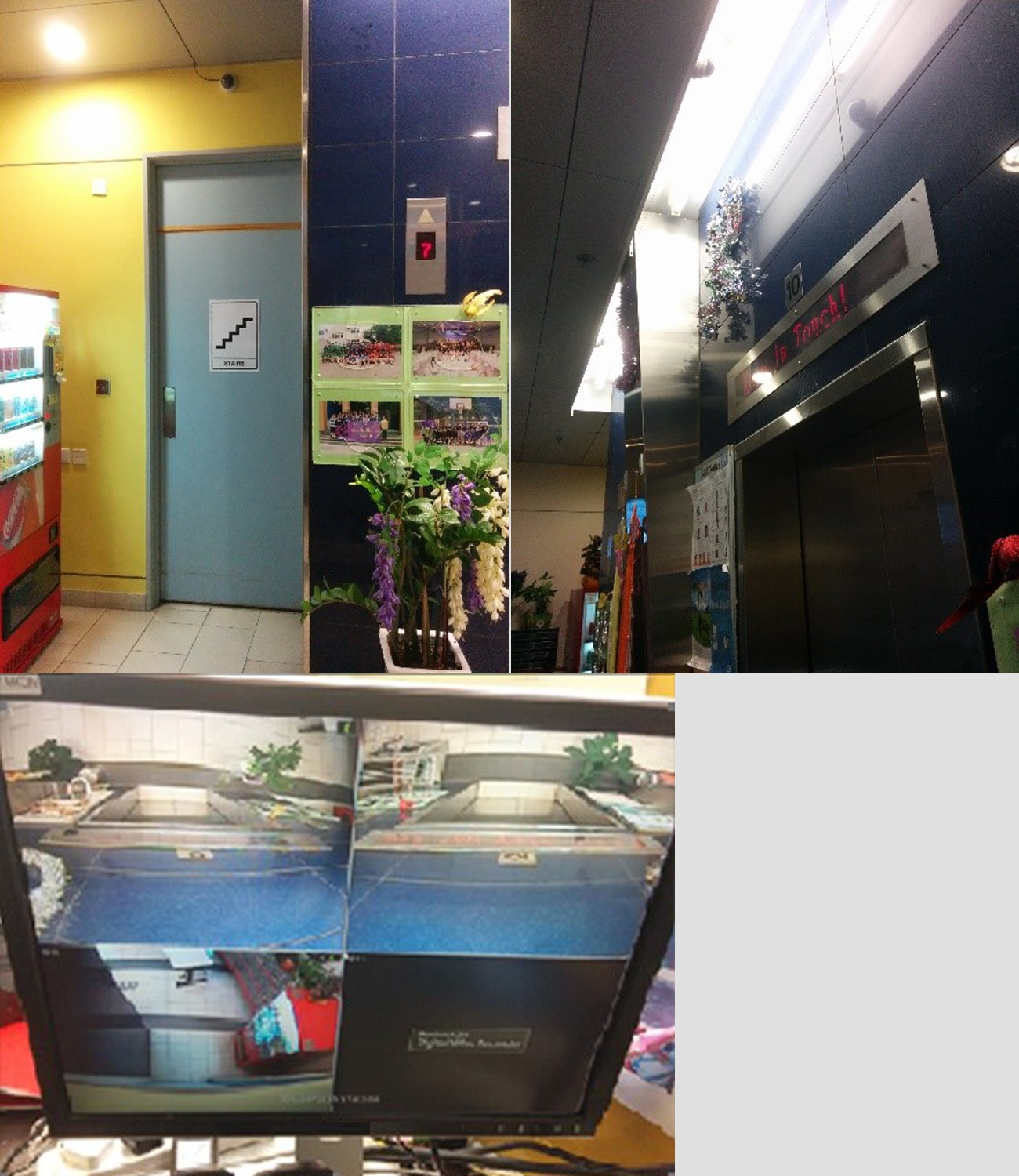

Supplement: S2 Fig — (TIF) [file pone.0225520.s003.tif]
